# Corrigendum

**DOI:** 10.1002/ueg2.12225

**Published:** 2022-04-02

In the article entitled “Ultrasonography in inflammatory bowel disease – So far we are?”,^1^ the authors' first names and surnames appeared in the wrong order. All author names have been corrected as follows:

Christian Maaser^1^

Giovanni Maconi^2^

Torsten Kucharzik^3^

Mariangela Allocca^4^

We apologize for this error.
